# Increased Circulating of CD54^high^CD181^low^ Neutrophils in Myelodysplastic Syndrome

**DOI:** 10.3389/fonc.2020.585216

**Published:** 2021-01-11

**Authors:** Liyan Yang, Hongzhao Li, Yumei Liu, Xinyan Xie, Huiqin Zhang, Haiyue Niu, Zonghong Shao, Limin Xing, Huaquan Wang

**Affiliations:** Department of Hematology, General Hospital, Tianjin Medical University, Tianjin, China

**Keywords:** myelodysplastic syndromes, neutrophils, immunity, CD54/ICAM-1, CD181

## Abstract

Myelodysplastic syndromes (MDSs) are a group of heterogeneous hematopoietic stem/progenitor cells clonal diseases, characteristic features with myeloid dysplasia, leading to abnormality of neutrophils. Recent studied have showed that neutrophils act not only as professional killers, but also as regulators of innate and adaptive immune in infection and inflammatory condition. The CD54^high^CD181^low^ neutrophils are a kind of reverse-transmigrated neutrophils characterized proinflammatory phenotype. We investigated the frequency and functional properties of circulating CD54^high^CD181^low^ neutrophils in patients with untreated MDS. Frequency of CD54^high^CD181^low^ neutrophils was significantly increased in MDS patients and related to the severity of the disease. Furthermore, CD54^high^CD181^low^ neutrophils suppressed CD8+ T cells functions *in vitro*. CD54^high^CD181^low^ neutrophils lead to upregulation of PD1 on CD8+ T cells. Higher CD54^high^CD181^low^ neutrophils were related to poor prognosis and more infections. The frequency of CD54^high^CD181^low^ neutrophils decreased in high risk MDS patients who had response after treatment with decitabine. Overall, we identified CD54^high^CD181^low^ neutrophils expanded in MDS. The exact mechanisms of increased CD54^high^CD181^low^ neutrophils and its effect on immune function remain to be elucidated.

## Introduction

Myelodysplastic syndrome (MDS) is a malignant clonal hematopoietic stem cell disease that is heterogeneous (1). It is characterized by malignant clonal hematopoietic stem cells, abnormal development of progenitor cells, ineffective hematopoiesis, and genetic instability, which makes it easy to transform into acute myeloid leukemia (AML) (2, 3).

Over the past decade, there has been increasing evidence of phenotype heterogeneity and functional diversity of neutrophils. In addition to their antibacterial functions, neutrophils appear as decision makers in innate and acquired immune responses. These findings open the door to understanding the role of neutrophils in homeostatic and pathogenic immune processes (4, 5). The differentiation of neutrophils into different functional subpopulations requires these cells to respond to environmental signals, synthesize and release a series of new proteins. Neutrophils resynthesize and release proteins that affected by epigenetics and regulatory genes (6).

Studies have shown that neutrophils have the ability to migrate out of the blood vessel and then return to the blood vessel, challenging the traditional concept of unidirectional neutrophil migration. The phenotype of neutrophils in the circulating pool is CD54^low^CD181^high^, and the phenotype in tissues is CD54^low^CD181^low^ *in vitro*. The phenotype of reverse-transmigrated neutrophils is CD54^high^CD181^low^, which is different from that in tissues and blood. These cells have a longer lifespan and more reactive oxygen species (ROS) production than circulating neutrophils (4). Reverse-transmigrated neutrophils exhibited a proinflammatory phenotype characterized by a high CD54 expression, and cannot transmigrate again into the tissue (6).

In the present study, we investigated the level of circulating CD54^high^CD181^low^ neutrophils in patients with MDS and evaluated the association between the neutrophils and T cells in MDS.

## Methods

### Patient Characteristics

From September 2016 to June 2019, a total of 37 newly diagnosed MDS patients in the Hematology Department of the General Hospital of Tianjin Medical University were enrolled in the study. The study included 22 males and 15 females with a median age of 61 years (range 27–79 years) (details in **Table 1**). The patients were divided into two groups based on the revised International Prognostic Scoring System (IPSS-R), the relative low risk group (IPSS-R score less than or equal to 3.5, n=18) and the relative high risk group (IPSS-R score more than 3.5, n=19). The low risk MDS patients were treated with Recombinant Human Erythropoietin (Sansheng, China) and lemalidomide (BeiGene, China) (only for 5q- patient). The high risk MDS patients were treated with decitabine (Janssen, China).

**Table 1:** The characteristics of myelodysplastic syndrome (MDS) patients.

case	sex/age	diagnosis	cytogenetics	IPSS-R
1	Male/63	MLD	46,XY	Very Low
2	Male/34	RS	46,XY	Very Low
3	Male/67	EB2	46,XY	Very high
4	Female/62	5q-	5q-	Low
5	Male/42	RS	46,XY,del20q11	Low
6	Female/47	RS	46,XX	Low
7	Male/62	SLD	46,XY,13q+	Low
8	Female/49	RS	46,XX	low
9	Male/50	EB1	46,XX	Int
10	Male/50	MLD	47,XY,+8/46,XY	Int
11	Male/65	MLD	46,XY,del17q31	Int
12	Female/61	MLD	46,XX	Int
13	Male/46	MLD	46,XY,-2,-12,+mar,19+,9P+	Int
14	Male/70	EB1	46,XY	Int
15	Female/67	MLD	46,XX	Int
16	Male/61	MLD	45-46,XY,21p+	Int
17	Male/71	MLD	46,XY	Int
18	Female/56	MLD	17P+, +8	Int
19	Male/58	MLD	46,XY	Int
20	Male/48	MLD	46,XY	Int
21	Male/58	EB2	46,XY	High
22	Female/73	EB2	46,XX	High
23	Male/61	EB2	46,XY	High
24	Female/64	EB2	46,XX	High
25	Female/59	EB1	46,XY,13q+	High
26	Male/62	EB2	46,XY	High
27	Male/38	EB2	46,XY	High
28	Female/70	EB2	46,XX	High
29	Female/69	EB2	46,XX	high
30	Male/30	EB2	47,XY,+8/46,XY	High
31	Female/79	EB2	45,XX,-7	Very High
32	Female/29	EB2	20q-,5q-,7q-	Very High
33	Male/68	EB2	46,XY,+8/45,XY+8,-6,-7	Very High
34	Female/76	EB2	del5q33,del5q31,del7q311,del7q3	Very High
35	Female/77	EB2	45,XX,-5,-2,45,XX,+mar,-5,3P-	Very High
36	Male/27	EB2	3p+,-18,+mar	Very High
37	Male/60	EB2	45,XY,-7	Very High

Twenty-three healthy people were selected as controls in this study, including 13 men and 10 women with a median age of 52 (range 24–74 years).

The study was approved by the Ethics Committee of the General Hospital of Tianjin Medical University. Informed written consents have been obtained from all patients and control groups or their guardians according to the Helsinki Declaration.

### CD54^high^CD181^low^ Neutrophils With Flow Cytometric Analysis

Heparin anticoagulant sterile tube were used to collect peripheral blood samples 5 ml from MDS patients and healthy controls. We used cells’ SSC/FSC to divide peripheral blood mononuclear cells into three subgroups, namely, lymphocytes, monocytes, and granulocytes. The CD 33 positive and CD 11b positive cells were defined neutrophils. The number of CD54^high^CD181^low^ neutrophils were measured by FCM assay. FITC-CD181, APC-CD11b, PE-CD54, and Cy7-CD33 monoclonal antibodies were purchased from BD Biosciences, USA. Data acquisition and analysis were performed using a FACS-Calibur flow cytometer (BD Biosciences, USA) and Cell Quest software (Becton Dickinson, version 3.1).

### CD54^high^CD181^low^ Neutrophils and CD8+ Cells Isolation

CD8 positive T cells were purified using CD8 MicroBeads isolation kit for human (No: 130-045-201, Miltenyl Biotec; Bergisch Gladbach, Germany) according manufacturer’s operating instructions. CD54^high^CD181^low^ neutrophils were sorted using FITC-CD181, APC-CD11b, PE-CD54, and Cy7-CD33 monoclonal antibodies by FACS-Aria (BD Biosciences, USA)(purity >95%).

### Coculture Experiments

CD54^high^CD181^low^ neutrophils and CD8+ T cells were coculture activating anti-CD2, -CD3, and -CD28 bead-coupled antibodies (Miltenyi Biotec, Bergisch Gladbach, Germany). The concentration of CD54^high^CD181^low^ neutrophils is 0,1×10^5^/ml and 2×10^5^/ml. The concentration of CD8+ T cells is 5×10^4^/ml.

### T-Cell Proliferation Assay

Proliferation of CD8+ T cells was assessed by Cell counting KIT-8 assay (CCK-8, Beyotime Biotechnology, China) and compared with stimulated T cells alone (set as 100% T-cell proliferation). CD8+ T cells were seeded on a 96-well plate at a density of 1 × 10^4^ with DMEM supplemented and 10% FBS. CD54^high^CD181^low^ neutrophils were added in experimental groups, and not in control group. After 3 days, 10 µl CCK-8 solution was added for an hour and its absorbance was measured with a microplate reader (Biotek Instruments Inc, Winooski, USA) at a wavelength of 450 nm.

### Lactate Dehydrogenase Measure

After coculture, the supernatants were collected and the levels of lactate dehydrogenase (LDH) in supernatant were detected by Roche Biochemistry Analyzer (Roche, Switzerland).

### Perforin and Granzyme With Flow Cytometric Analysis

Perforin and granzyme secreted by CD8+ T cells were measured using perforin-PE and granzyme-APC monoantibody (BD Biosciences, USA) by FACS-Calibur flow cytometer (BD Biosciences, USA). Briefly, after coculture, the cells were collected and labeled with CD8-FITC (BD Biosciences, USA). After cell fixation and permeabilization, perforin-PE and granzyme-B-APC were added.

### PD1 With Flow Cytometric Analysis

PD1 on CD8+ T cells were measured using PD1-APC monoantibody (BD Biosciences, USA) by FACS-Calibur flow cytometer (BD Biosciences, USA). Briefly, after coculture, the cells were collected and labeled with CD8-FITC (BD Biosciences, USA) and PD1-APC monoantibody.

### Statistical Analysis

Result analysis was performed with the GraphPad Prism 8.0 program (GraphPad Software, Inc. San Diego, CA). Data obeyed normal distribution were presented as means ± SD and multiple group comparisons were performed by using one-way analysis of variance (ANOVA). The analysis of correlation was performed by linear regression. The survival analysis was performed by Log-Rank test. The ratio of transformation to AML and infection were performed by Fisher’s exact test. The index changes before and after treatment were performed by paired t test. A P value of <0.05 was considered statistically significant.

## Results

### Circulating CD54^high^CD181^low^ Neutrophils Increased in Untreated MDS and Correlated With High Risk According to IPSS-R

**Figure 1:**
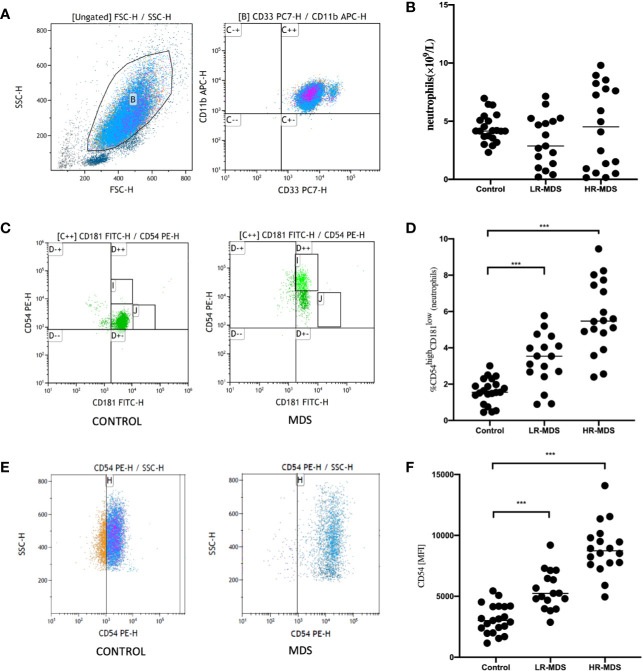
Increased frequency of CD54^high^CD181^low^ neutrophils cells in the peripheral blood of new diagnosis myelodysplastic syndromes (MDS) patients. **(A, B)** Frequency of peripheral blood neutrophils were compared between 23 healthy controls (HC) and 37 new diagnosis MDS patients. FSC and SSC were used to gate neutrophils. CD33 positive and CD11b positive were used to confirm neutrophils (C++). **(C, D)** Representative dot plots from flow cytometric (FACS) analyses showing the CD54^high^CD181^low^ cell frequency among peripheral blood neutrophils obtained from HC (n = 23) and MDS patients (n = 37) (I represents CD54^high^CD181^low^ neutrophils). **(E, F)** Mean fluorescence index (MFI) levels of CD54 on the CD33 and CD11b neutrophils was compared between HC (n = 23) and MDS patients (n = 37). The bars represent the standard error of the mean. ***P < 0.001.

Frequency of CD54^high^CD181^low^ neutrophils among peripheral blood were significantly increased in MDS patients (3.33 ± 1.42% in LR-MDS and 5.71 ± 1.97% in HR-MDS) as compared with healthy controls (1.61 ± 0.70%) (**Figures 1C, D**). The mean fluorescence index (MFI) levels of CD54 on the CD33+CD11b+ neutrophils were significantly increased in MDS patients (5510 ± 1590 in LR-MDS and 8906 ± 2103 in HR-MDS) as compared with healthy controls (3182 ± 1187) (**Figures 1E, F**). But the quantity of neutrophils among peripheral blood were not significantly different among MDS patients and healthy controls (4.49 ± 3.55×10^9^/L, 3.29 ± 2.19×10^9^/L and 4.42 ± 1.25×10^9^/L, respectively) (**Figures 1A, B**). Therefore, the increase of CD54^high^CD181^low^ neutrophils is not due to granulocytosis.

### CD54^high^CD181^low^ Neutrophils From MDS Patients Suppress T Cells Functions

**Figure 2:**
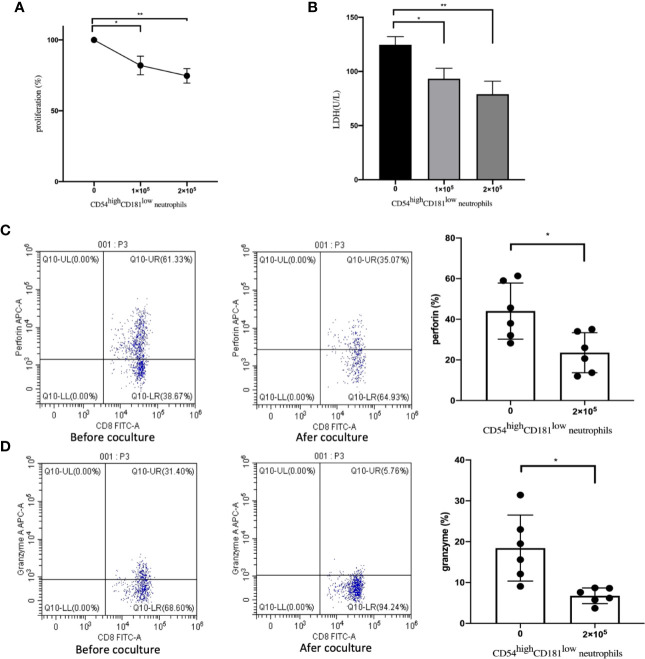
Impact of CD54^high^CD181^low^ neutrophils from myelodysplastic syndromes (MDS) patients on T cells. **(A)** The dose-dependent suppressive activity of FACS-sorted CD54^high^CD181^low^ neutrophils from MDS patients (n=6) was evaluated in coculture experiments with autologous T cells activated by means of anti-CD2, -CD3, and -CD28 microbeads. Proliferation of T cells was assessed after 5 days by CCK-8 assay and compared with stimulated T cells alone (set as 100% T-cell proliferation). **(B)** The levels of lactate dehydrogenase (LDH) in supernatant of coculture experiments with CD54^high^CD181^low^ neutrophils, CD3+ T cells and effector cells. **(C)** The perforin of CD3+CD8+ T cells before and after coculture with CD54^high^CD181^low^ neutrophils from the peripheral blood of MDS patients. **(D)** The granzyme B of CD3+CD8+ T cells before and after coculture with CD54^high^CD181^low^ neutrophils from the peripheral blood of MDS patients. The bars represent the standard error of the mean. *P < 0.05; **P < 0.01.

In the following experiment, we sorted CD54^high^CD181^low^ neutrophils and CD8+ T cells from MDS patients using FACS and CD8 MicroBeads. In order to prove the *in vitro* suppressive capacity of CD54^high^CD181^low^ neutrophils on T cell, proliferation of sorted T cells was measured after bead stimulated. It was found that CD54^high^CD181^low^ neutrophils cells suppressed T cell proliferation significantly with in a dose-dependent manner (**Figure 2A**). The levels of lactate dehydrogenase (LDH) in supernatant of coculture experiments with CD3+ T cells and effector cells decreased significantly when CD54^high^CD181^low^ neutrophils was added (**Figure 2B**). The level of perforin and granzyme secreted by T cells were decreased significantly while co-culturing with CD54^high^CD181^low^ neutrophils (44.04 ± 5.64 *vs.* 23.58 ± 4.03 and 18.45 ± 3.30 *vs.* 6.76 ± 0.79) (**Figures 2C, D**).

### CD54^high^CD181^low^ Neutrophils Lead to Upregulation of PD1 Expression on CD8+ T Cells

**Figure 3:**
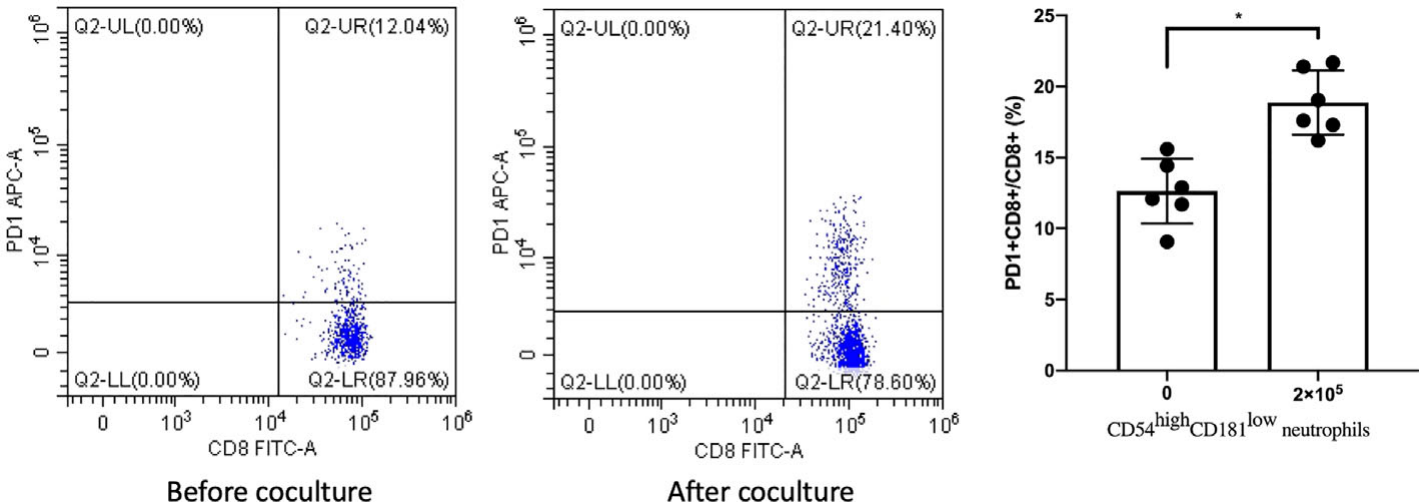
CD54^high^CD181^low^ neutrophils from myelodysplastic syndromes (MDS) patients (n = 6) lead to upregulation of PD1 expression on CD8+ T cells. The bars represent the standard error of the mean. *P < 0.05.

Next, we measured the levels of PD1 on CD8+T cells using FACS before and after co-culturing with CD54^high^CD181^low^ neutrophils. We found that the expression of PD1 on CD8+T cells from MDS patients was increased significantly after co-culturing with CD54^high^CD181^low^ neutrophils (12.63 ± 2.28 vs. 18.87 ± 2.31) (**Figure 3**).

### Higher CD54^high^CD181^low^ Neutrophils Related With Poor Prognosis and More Infection

**Figure 4:**
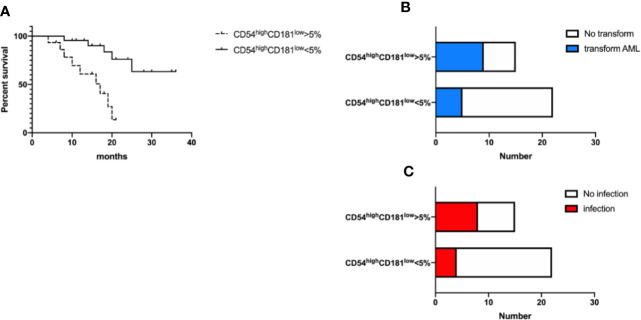
CD54^high^CD181^low^ neutrophils are related to the prognosis and infection of myelodysplastic syndromes (MDS) patients. **(A)** The frequency of CD54^high^CD181^low^ neutrophils is related with overall survival of MDS patients. *P* < 0.05 **(B)** The ratio of acute myeloid leukemia transformation was higher in higher frequency of CD54^high^CD181^low^ neutrophils group. *P*<0.05 **(C)** The incidence of infection was higher in higher frequency of CD54^high^CD181^low^ neutrophils group at diagnosis time. *P* < 0.05.

In order to verify the impact of CD54^high^CD181^low^ neutrophils on survival, acute myeloid leukemia transformation and infection, we divided MDS patients into two groups based on whether CD54^high^CD181^low^ neutrophils were greater than 5%, a high-proportion group (n=15) and a low-proportion group (n=22). The median follow-up time was 17 months (range 4–36 months). The median overall survival of MDS patients with more than 5 percent CD54^high^CD181^low^ neutrophils was 17 months. The median overall survival of MDS patients with less than 5 percent CD54^high^CD181^low^ neutrophils was not reach during the follow-up time. The survival of higher CD54^high^CD181^low^ neutrophils MDS patients was shorter than that of lower CD54^high^CD181^low^ neutrophils patients (*P*<0.05) (**Figure 4A**).

Nine patients (60%) transformed to acute myeloid leukemia in higher CD54^high^CD181^low^ neutrophils MDS group during following-up period, while only five patients (22.7%) transformed in lower group (*P*<0.05) (**Figure 4B**). Transformed acute myeloid leukemia included five acute monocytic leukemia (M5) cases, three acute myelomonocytic leukemia (M4) cases and one pure erythroid leukemia (M6) case in higher group, and three M5 cases and two M4 cases in lower group.

Eight patients (53.3%) had infection at the time of new diagnosis in higher CD54^high^CD181^low^ neutrophils MDS group, but only four patients (18.2%) had infection in lower group (**Figure 4C**). The most common sites of infection are the lungs (6 cases), upper respiratory tract (two cases), skin (one case), mouth (one case), perianal (one case), and blood (one case).

### The Frequency of CD54^high^CD181^low^ Neutrophils Decreased in High Risk MDS Patients Who Had Response

**Figure 5:**
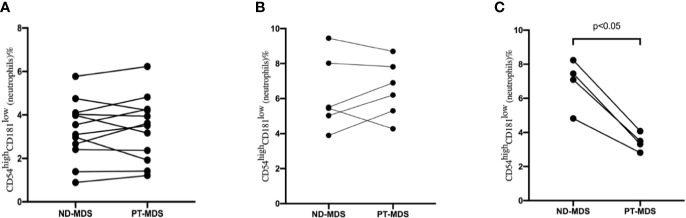
The change of CD54^high^CD181^low^ neutrophils from myelodysplastic syndromes (MDS) patients at new diagnosis (ND) and post-therapy (PT). **(A)** Low risk MDS patient (n = 12). *P* > 0.05. **(B)** high risk MDS patients without good response (CR+PR) (N = 6). *P* > 0.05. **(C)** high risk MDS patients with good response (CR+PR) (N = 4). *P* < 0.05.

Among the 37 MDS patients, 22 patients had the data of CD54^high^CD181^low^ neutrophils at new diagnosis and after treatment, including 12 in the low-risk group and 10 in the high-risk group. The median interval time is 3 months (range 1–6 months). The frequency of CD54^high^CD181^low^ neutrophils in the relatively low-risk group did not change significantly before and after treatment (*P*>0.05) (**Figure 5A**). But the frequency of CD54^high^CD181^low^ neutrophils in the high-risk group who had good responses (complete response or partial response) after treatment was decreased significantly (*P*<0.05) (**Figure 5B**). However, the frequency of CD54^high^CD181^low^ neutrophils did not change significantly in patients with high-risk MDS who did not respond to treatment (*P*>0.05) (**Figure 5C**).

## Discussion

MDS is a clonal hematopoietic abnormality disease. Most MDS patients have aberrant neutrophil development. Aberrantly developed neutrophils not only have abnormal quantity, but can also lead to abnormal quality. The abnormality of neutrophils not only leads to an increased chance of infection, but may also affect the immune function of patients. In the past decade, MDS clone leading to abnormal pro-inflammatory signaling and innate immune activation in bone marrow microenvironment were identified as key pathogenic driver factors (7, 8).

Recent evidences indicate that tumors regulate neutrophil function to support tumor growth and development. Tumor associated neutrophils are an important part of the tumor microenvironment and play an active role in tumor occurrence, progression and metastasis (9).

Here, we found that circulating CD54^high^CD181^low^ neutrophils increased in untreated MDS and correlated with high risk according to IPSS-R. CD54, also known as ICAM -1, is a transmembrane glycoprotein of the immunoglobulin superfamily, involved in tumor cell immune regulation, angiogenesis, invasion and distant metastasis. Some studies have proved that CD54 is abnormally highly expressed in a variety of malignant tumors, which promotes the occurrence and development of tumors and affects their prognosis. Several studies have shown that CD54 is highly expressed in CD34+ cells and CD8+ lymphocytes in MDS patients, and the soluble CD54 level in plasma of MDS is also significantly increased, which is an important indicator of cytopenia, dysplasia, and disease progression to AML (10–14).

CD181, also known as CXCR1, is mainly expressed on the surface of neutrophils in the normal body, when foreign pathogens enter invasion, causing inflammation in the tissues, the inflammatory tissue releases IL-8 and other chemokines, and binds to CD181 on the surface of neutrophils through blood circulation, thereby migrating neutrophils to the site of inflammation to engulf and kill pathogens (15). Fuhler et al. have demonstrate decreased CD181-induced neutrophil chemotaxis in MDS patients (16).

We found that circulating CD54^high^CD181^low^ neutrophils from MDS patients suppress T cells functions. Our previous study has shown that myeloid-derived suppressor cells (MDSCs) were increased in MDS patients, and related poor clinical outcome. MDSCs could suppress T-cell mediated immune functions (17, 18). MDSCs are a group of immature myeloid cells derived from bone marrow, including monocytic and granulocytic MDSCs. Recently, a few studies have demonstrated that mature neutrophils could exert MDSC activity suppressing T cells functions (19, 20). Our research indicated that not only immature myeloid cells can inhibit T lymphocytes, but mature granulocytes CD54^high^CD181^low^ neutrophils can also inhibit T lymphocytes. Only activated neutrophils with ROS can suppress T-cell functions. CD54^high^CD181^low^ neutrophils can produce more ROS and have a longer lifespan. So CD54^high^CD181^low^ neutrophils could exert MDSC suppressive activity about T cells. Silzle et al. (21) reported that lymphopenia was associated with increased mortality in patients with MDS, and confirmed that innate and adaptive immune system changed in MDS patients. The mechanism of impaired lymphatic homeostasis balance included MDS clonal cell, bone marrow microenvironments and genetic mutations.

Our results showed that CD54^high^CD181^low^ neutrophils lead to upregulation of PD1 expression on CD8+ T cells. PD1, the full name is programmed cell death protein 1, is majorly expressed on active T lymphocytes, and plays an essential role in balancing immune tolerance, protective immunity and homeostasis. In patients with chronic infection or tumors, PD1 over-express can inhibit T cell effector functions, promote T cell exhaustion and contribute to adaptive resistance. Tumor patients often have physiological stress, for instance, hypoxia and nutrient deprivation. Cancer cells can hijack immune suppression (22). We speculate, in patients with MDS, due to the persistent presence of hypoxia and an inflammatory state, CD54^high^CD181^low^ neutrophils promote over-expression of PD1 on CD8+ T cells and inhibit anti-tumor immunity of CD8+ T cells.

Does elevated CD54^high^CD181^low^ neutrophils affect overall survival and acute leukemia transformation? We found that the overall survival time of patients with higher CD54^high^CD181^low^ neutrophils group was significantly shorter than that of patients with lower proportion. This shows that a high proportion of CD54^high^CD181^low^ neutrophils is a factor of poor prognosis. The rate of transformation to acute leukemia was also significantly higher in the high proportion of CD54^high^CD181^low^ neutrophils group than in the low-proportion group. Of course, most patients in the high proportion of CD54^high^CD181^low^ neutrophils group are patients in the relatively high-risk group, and this part of the patients themselves has a poor prognosis. But generally speaking, the high proportion of neutrophils indicates that patients have a higher chance of acute leukemia transformation and a shorter expected survival. AML transformation, infection and bleeding are the main causes of death in MDS patients. The degree of neutropenia was significantly correlated with mortality (23). The relationship between different phenotypes of neutrophils and prognosis needs to be further studied (24, 25). We also found that the risk of infection in the group with a high proportion of CD54^high^CD181^low^ neutrophils was higher at the new diagnosis time. Infection is the leading cause of death in MDS patients, which mainly attributed to decreased counts and qualitative defects of neutrophil (26–28).

In order to study the effect of CD54^high^CD181^low^ neutrophils on the therapeutic effect, we examined the changes of neutrophils before and after treatment. We found that for patients in the low proportion group, the frequency of CD54^high^CD181^low^ neutrophils did not change significantly before and after treatment. This may be related to the fact that the main clinical manifestation of patients in the relatively low-risk group is anemia. Blood transfusion and rhEPO therapy were used had little effect on neutrophils. The frequency of CD54^high^CD181^low^ neutrophils decreased in patients who had achieved good response after decitabine treatment. But the frequency of CD54^high^CD181^low^ neutrophils did not change significantly in patients who had no good response. This also indirectly proves that high CD54^high^CD181^low^ neutrophils are related to poor prognosis.

In conclusion, we found that CD54^high^CD181^low^ neutrophils increased in peripheral blood of MDS patients, and CD54^high^CD181^low^ neutrophils could suppress CD8+ T cells functions and upregulate the PD1 expression on CD8+ T cells. CD54^high^CD181^low^ neutrophils affect the prognosis and the chance of infection. We conclude CD54^high^CD181^low^ neutrophils may be involved in MDS pathogenesis and targeting strategies offer potential therapy for MDS.

## Data Availability Statement

The original contributions presented in the study are included in the article/**Supplementary Material**. Further inquiries can be directed to the corresponding authors.

## Ethics Statement

The studies involving human participants were reviewed and approved by The ethics committee of General Hospital Tianjin Medical University. The patients/participants provided their written informed consent to participate in this study.

## Author Contributions

LY, HL, and YL performed research and analyzed the data. HW design studies, ensure the correct analysis of the data and drafted the manuscript. XX, HZ, HN, LX, and ZS assisted in design research, oversaw data collection, and contributed to the writing of the manuscript. All authors contributed to the article and approved the submitted version.

## Funding

This project is partly supported by The National Natural Science Foundation of China (No. 81170472), Key Technology Research and Development Program of Tianjin China (18ZXDBSY00140), Application Bases and Advanced Technology Research Program of Tianjin China (No. 14JCYBJC27200, 09JCYBJC11200).

## Supplementary Material

The Supplementary Material for this article can be found online at: https://www.frontiersin.org/articles/10.3389/fonc.2020.585216/full#supplementary-material

## Conflict of Interest

The authors declare that the research was conducted in the absence of any commercial or financial relationships that could be construed as a potential conflict of interest.
