# Supplementary material for: Increased Circulating of CD54highCD181low Neutrophils in Myelodysplastic Syndrome
Source: Front Oncol. 2021 Jan 11;10:585216. doi: 10.3389/fonc.2020.585216 (PMC7830137; doi:10.3389/fonc.2020.585216)
Supplement: Supplementary file 1 [file DataSheet_1.docx]

***Sample collection, preparation, gate and analysis:***

Whole blood sample was obtained by standard venipuncture using disposable venous blood lancets and vacuum blood tubes. Five milliliter testing blood sample was then collected by heparin anticoagulant sterile tube.

FITC-CD181, APC-CD11b, PE-CD54, Cy7-CD33 and isotype controls monoclonal antibodies were purchased from BD Biosciences. All antibodies were titrated to saturating concentrations and the corresponding isotype controls were prepared. Diluted blood was stained and incubated in the dark at room temperature for 15-20 minutes. Flow cytometric measurements were performed within 30 minutes after the samples were obtained. Data acquisition and analysis were performed using a FACS-Calibur flow cytometer (BD Biosciences, USA) and Cell Quest software (Becton Dickinson, version 3.1).

We used cells’ forward scatter (FSC) and side scatter (SSC) to divide peripheral blood mononuclear cells into three subgroups, namely lymphocytes, monocytes, and granulocytes (B in figure 1).


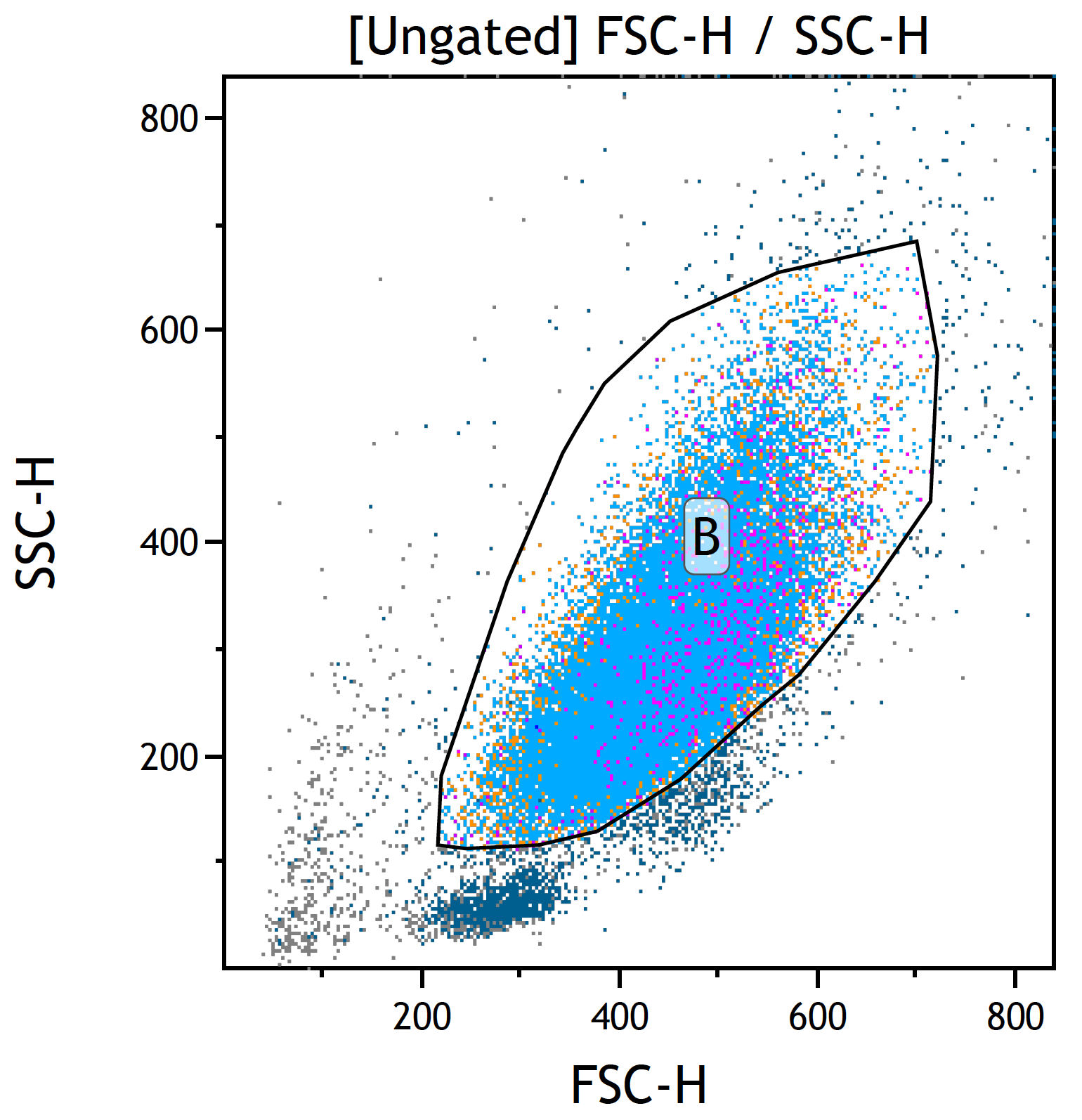


Figure 1 FSC and SSC of peripheral blood mononuclear cells

Then we gated the granulocytes (B in figure 1) and used CD 33 and CD 11b to define neutrophils (C++ in figure 2). Mature neutrophils express CD33 and CD11b.

Reference:

Arber D.A., Orazi A Hasserjian RP, et al. Introduction and overview of the classification of myeloid neoplasms. In: Swerdlow SH, Campo E, Harris NL, et al., eds. WHO classification of tumors of haematopoietic and lymphoid tissues. Lyon, France: International Agency for Research on Cancer, 2017: 16-27.


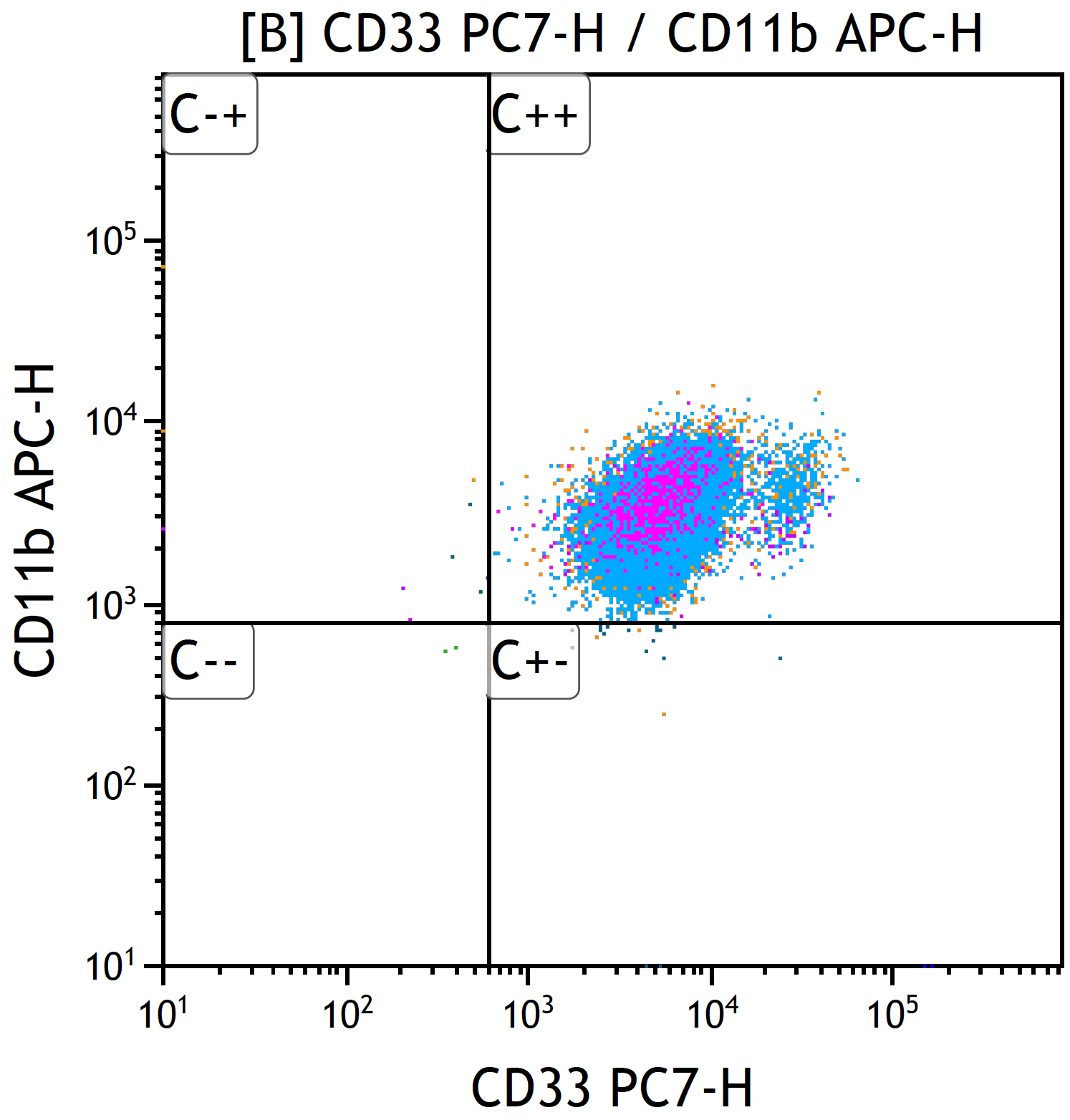


Figure 2 CD33 and CD11b of granulocytes

Next, we used CD54 and CD181 to measure the number of CD54^high^CD181^low^ neutrophils by FCM assay. I in D++ (figure 3) was the CD54^high^CD181^low^ neutrophils.


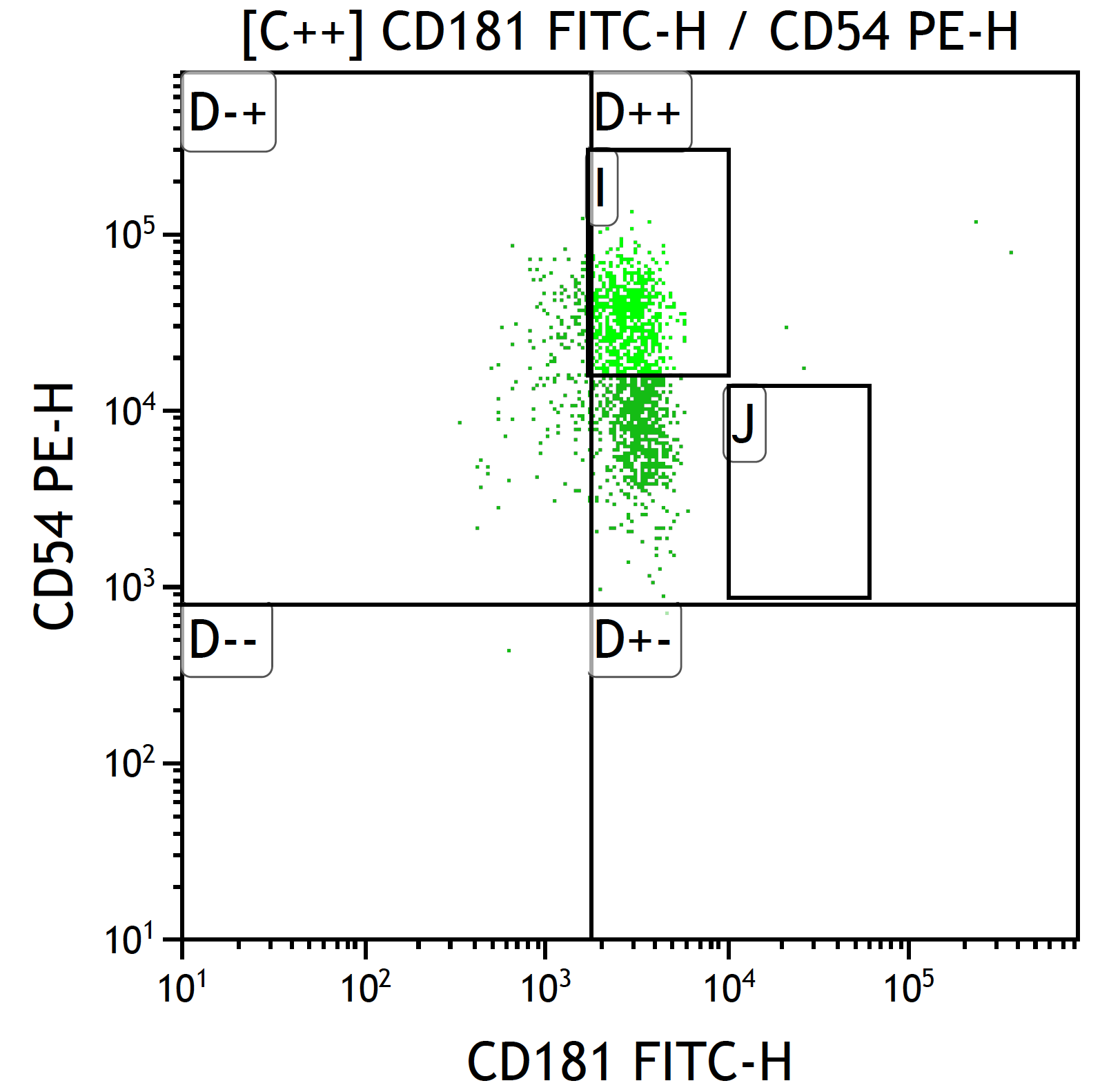


Figure 3 CD54 and CD181 of neutrophils
